# Current pharmacologic treatment of brain metastasis in non-small cell lung cancer

**DOI:** 10.1007/s10585-024-10276-4

**Published:** 2024-03-11

**Authors:** Takae Okuno, Takeshi Isobe, Yukari Tsubata

**Affiliations:** Division of Medical Oncology and Respiratory Medicine, Department of Internal Medicine, Shimane University Faculty of Medicine, 89-1, Enyacho, Izumo, Shimane 693-8501 Japan

**Keywords:** Non-small-cell lung cancer, Brain metastasis, Pharmacologic treatment, Molecular target inhibitor, Immune checkpoint inhibitor

## Abstract

Lung cancer is a type of cancer that can metastasize to the lungs, brain, bones, liver, adrenal glands, and other organs; however, the occurrence of brain metastases is the most common event. Symptoms of brain metastasis include motor dysfunction, mental dysfunction, seizures, headaches, nausea, and vomiting, and significantly reduce the quality of life of cancer patients. Brain metastases are a poor prognostic factor, and controlling them is extremely important for prolonging prognosis and improving the quality of life. Currently, local surgery and radiotherapy are recommended for their treatment. However, recently, cancer treatments using molecular-targeted drugs and immune checkpoint inhibitors have been introduced, which may also be effective against brain metastases. Therefore, it is necessary to determine whether local or systemic therapy is optimal for each case. In this review, we focus on recent findings regarding drug therapy in treating brain metastases from advanced non-small cell lung cancer.

## Introduction

Lung cancer tends to metastasize to the central nervous system (CNS) and is the most frequent metastatic brain tumor [1]. In non-small cell lung cancer (NSCLC), the percentage of brain metastases at initial diagnosis is approximately 10–30% and increases with the course of treatment [2–4]. Brain tumors are classified into general and local symptoms/signs, and patients often present with both. Generalized nonspecific symptoms include headaches, cognitive dysfunction, renal girdle changes, and gait disorders. Systemic symptoms are caused by increased intracranial pressure or direct impairment of cerebrospinal fluid (CSF) circulation due to tumor growth and surrounding edema. Headache may be accompanied by nausea and vomiting owing to increased intracranial pressure. Changes in personality may include amnesia (loss of interest and blunted affect) and withdrawal from social situations that mimic depression. The localized and unilateral findings include hemiparesis, speech impairment, and visual field defects. Unilateral symptoms such as hemiplegia are often subacute and progressive. Language impairment can be mistaken for confusion, and visual field defects often go unnoticed by patients. Epileptic seizures are another common symptom, occurring in approximately 25% of patients with brain metastasis [5]. These symptoms decrease quality of life (QOL) and often require emergency oncology services with medical oncologists, neurologists, and neuro-oncologists. The number and location of metastatic brain tumors, the patient's general condition, the overall cancer status, and the likelihood that the cancer can be treated are factors that determine the treatment, such as surgery, radiation, drug therapy, or a combination of these.

Brain metastasis is a poor prognostic factor, and its control may prolong the prognosis and improve QOL.

## Prognostic score for brain metastases

In 2008, Sperduto et al. [6] proposed a graded prognostic assessment (GPA) score based on multivariate analysis of 1960 patients with brain metastases from five randomized trials of the Radiation Therapy Oncology Group (RTOG) in the US. In addition, diagnosis-specific GPAs (DS-GPAs) for each cancer type were generated from the data of 4259 patients with brain metastases from five types of cancers (NSCLC, breast cancer, renal cell cancer, gastrointestinal cancer, and malignant melanoma) [7]. Subsequent analysis of the four DS-GPA factors for lung cancer (age, KPS, number of extracranial and brain metastases), genetic mutation status (*EGFR*, *ALK*, *K-RAS*), smoking index, sex, race, histopathological grade, and total number of brain metastases revealed *EGFR* and *ALK* to be new prognostic factors in patients with lung adenocarcinoma [8].

However, with the recent use of molecular-targeted agents and immune checkpoint inhibitors other than *EGFR* and *ALK* mutations, this score is insufficient for making treatment decisions. Therefore, it is necessary to determine the optimal treatment for brain metastases through consultation with each specialist based on the latest findings.

## Local and systemic treatment of brain metastases

Brain metastases can be treated with local therapy, such as surgery, radiotherapy, or systemic therapy with anticancer drugs; the type of therapy is determined based on histologic type, general condition of the patient, and size and number of brain metastases.

### Surgery

#### ASCO-SNO-ASTRO guidelines

The ASCO-SNO-ASTRO guidelines [9] state that patients with brain metastases should undergo surgery considering the following factors: (1) patients with brain metastases who are suspected of having undiagnosed primary cancer and are likely to benefit from surgery to confirm the diagnosis and remove the tumor, (2) patients with large tumors and mass effects who may also benefit from surgery, and (3) patients with multiple brain metastases and uncontrolled systemic disease who may benefit less from surgery unless the remaining disease can be controlled using other means. This recommendation is based on expert consensus and the quality of the evidence is mixed; therefore, it is considered a medium-strength recommendation.

#### EANO-ESMO guidelines

The patients who benefited from surgery were generally identical to those described in the ASCO-SNO-ASTRO guidelines. For single brain metastases, the EANO-ESMO guidelines [10] state that the therapeutic value of neurosurgical resection in patients with controlled systemic disease is undisputed and that, more than any other intervention, it allows for rapid steroid tapering and optimizes the therapeutic effect of subsequent therapies, especially immune checkpoint inhibitors. The ASCO-SNO-ASTRO guidelines state that surgery may be reasonable for patients with large tumors with mass effects, but less so for patients with small metastases that can be treated noninvasively, such as by stereotactic radiosurgery (SRS). The EANO-ESMO guidelines state that surgical intervention may improve outcomes in patients with multiple brain metastases if complete gross resection is possible. It is also stated that surgery should be considered for immediate effect in cases where large brain metastases (> 3 cm in diameter) are located in a specific brain region and cause increased intracranial pressure and neurological dysfunction.

#### NCCN guidelines

The NCCN guidelines [11] state that the purpose of surgery is to obtain tissue for diagnosis, reduce the mass effect, and reduce edema, similar to the two previous guidelines. They stated that SRS is an excellent minimally invasive ablation treatment option that avoids the risk of surgery-related complications and is generally preferred over surgery for small asymptomatic lesions that do not require surgery or are not surgically accessible.

### Radiation therapy

**Fig. 1:**
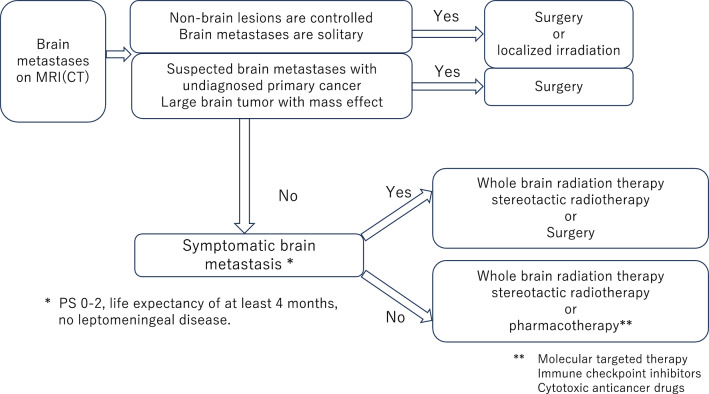
Treatment strategies according to the status of brain metastases

These guidelines recommend that patients with symptomatic brain metastases should be treated with local therapy (radiosurgery and/or both) regardless of whether systemic therapy is offered. Deferral of local therapy is not recommended, even in patients with asymptomatic brain metastases. However, deferral of local therapy is acceptable for patients with asymptomatic brain metastases receiving EGFR-TKIs (icotinib or osimertinib), ALK-TKIs (alectinib, ceritinib, and brigatinib), and platinum + pemetrexed + pembrolizumab in PD-L1 positive patients. The decision to defer local therapy should be based on a multidisciplinary (neuro-oncology, medical oncology, neurosurgery, and radiation oncology) discussion of the potential benefits and harms that patients may experience. A schematic diagram of local therapy (surgery or radiation therapy) is shown in Fig. 1. NCCN guidelines also state that there are increasing numbers of systemic treatment options with demonstrated activity in the brain, it is now reasonable to treat patients with asymptomatic brain metastases with systemic therapy upfront instead of upfront SRS or WBRT. The EANO-ESMO guideline do not explicitly state the possibility that radiotherapy for brain metastases can be postponed by effective systemic therapy.

## Systemic therapy

Many brain metastases are resistant to treatment because they are insensitive to anticancer drugs and acquired drug resistance. Additionally, their inability to penetrate the blood–brain barrier (BBB) can result in resistance to treatment [12]. Local therapies such as surgery or radiotherapy are recommended for symptomatic brain metastases. However, with the recent increase in the number of drugs effective against brain metastases, it is now reasonable to administer systemic therapy before local therapy for asymptomatic brain metastases. Other reasons for systemic therapy over radiation therapy are as follows: (1) adjuvant radiation therapy after surgery improves intracranial local control but does not affect overall survival, and the length of time patients remain functionally independent [13]. (2) Patients who develop grade 3 or atrophic leukoencephalopathy after radiosurgery have a significantly worse clinical status and quality of life. More than 20% of patients may develop radiation necrosis after radiosurgery, and steroids are required to develop and control their symptoms [14]. (3) Radiotherapy can cause cognitive impairment and permanent disability, which can affect neurocognitive function and severely affect daily life [15]. When systemic therapy precedes local therapy for brain metastases, continuous CNS surveillance with head magnetic resonance imaging (MRI) is essential for prompt intervention in cases of disease progression or inadequate response. In this review, we discuss drug therapies for NSCLC.

### Molecular targeted drugs

#### EGFR tyrosine kinase inhibitors

##### Osimertinib

According to a 2013 meta-analysis of epidermal expression frequencies in NSCLC, the frequencies (*EGFR*) gene mutations are 47.9% and 19.2% in adenocarcinomas and 4.6% and 3.3% in squamous cell carcinomas in Asians and Westerners, respectively, according to a 2013 meta-analysis (mut MAP) of *EGFR* mutation expression frequency in NSCLC [16]. Several EGFR tyrosine kinase inhibitors (EGFR-TKIs) are effective against *EGFR*-mutated lung cancer, among which third-generation osimertinib is widely used. In a phase III trial (FLAURA) comparing osimertinib with first-line treatment first-generation EGFR-TKIs (gefitinib or erlotinib), osimertinib significantly prolonged progression-free survival (PFS), the primary endpoint, and became the standard treatment (osimertinib 18.9 months [95% CI 15.2–21.4] vs first-generation EGFR-TKI 10.2 months [95% CI 9.6–11.1]; HR 0.46; 95% CI 0.37–0.57; *p* < 0.001) [17]. It also showed an overall survival (OS) benefit (osimertinib 38.6 months [95% CI 34.5–41.8] vs first-generation EGFR-TKI 31.8 months [95% CI 26.6–36.0]; HR 0.80; 95% CI 0.64–1.00; *p* = 0.046) [18], and was recommended as a first-line treatment. In the FLAURA trial, 20.9% (116/556 patients) had CNS metastases at enrollment; and median PFS for patients with CNS metastases was longer in the osimertinib group than in the first-generation EGFR-TKI group (15.2 months vs 9.6 months; HR 0.47; 95% CI 0.30–0.74; *p* < 0.001). The progression of CNS metastasis was 6% (17/279) in the osimertinib group and 15% (42/277) in the first-generation EGFR-TKI group, regardless of CNS metastasis at enrollment, suggesting that osimertinib is effective in patients with brain metastases [17]. The FLAURA trial also analyzed intracranial lesion control in patients with asymptomatic brain metastases. In patients with measurable and/or non-measurable CNS lesions, CNS-PFS was not achieved with osimertinib (95% CI 16.5 months to incalculable) and was 13.9 months with first-generation EGFR-TKIs (95% CI 8.3 months to incalculable) (HR 0.48; 95% CI 0.26–0.86; *p* = 0.014), indicating that osimertinib significantly prolonged CSN-PFS compared to first-generation EGFR-TKIs. The CNS objective response rate (ORR) in the population with one or more measurable CNS lesions was 91% for osimertinib and 68% for first-generation EGFR-TKIs (odds ratio 4.6; 95% CI 0.9–34.9; *p* = 0.066), showing a good intracranial disease control rate [19].

The AURA3 trial compared osimertinib with pemetrexed/platinum-based therapy in 419 patients with T790M mutation-positive advanced NSCLC, who progressed after treatment with first-generation EGFR-TKIs. Patients with CNS metastases accounted for 34.4% (144/419), and osimertinib was superior in PFS among patients with CNS metastases (8.5 months vs 4.2 months; HR 0.32; 95% CI 0.21–0.49) [20]. In a study of 46 patients with CNS metastases and one or more measurable lesions, the CNS-ORR was 70% for osimertinib (21/30 patients; 95% CI 51–85%) and 31% for pemetrexed/platinum (5/16 patients; 95% CI 11–59%). the osimertinib group was significantly higher (odds ratio, 5.13; 95% CI 1.44–20.64; *p* = 0.015). CNS-duration of response (DoR) was 8.9 months (95% CI 4.3 months–not calculated) vs 5.7 months (95% CI 4.4–5.7), CNS-PFS was 11.7 months vs 5.6 months (HR 0.32; 95% CI 0.15–0.69; *p* = 0.004), and osimertinib showed better CNS efficacy than pemetrexed/platinum in T790M-positive advanced NSCLC [21].

Osimertinib has been reported to have a higher rate of CNS penetration than first-generation EGFR-TKIs. In preclinical studies on brain penetration and activity of EGFR-TKIs in various animal models, osimertinib showed greater penetration of the mouse BBB than gefitinib, rociletinib (CO-1686), and afatinib. In addition, osimertinib caused sustained tumor shrinkage at clinical doses in an *EGFR*-mutated mouse model of PC9 brain metastasis. In addition, under positron emission tomography microdosing conditions, [11C]osimertinib showed significantly higher exposure in the cynomolgus monkey brain than [11C]rociletinib and [11C]gefitinib [22]. Osimertinib is the weakest substrate of human BBB efflux transporters in vitro. The in vivo rat-free brain to free plasma ratio (Kpuu) showed that osimertinib had the highest BBB penetration compared to other EGFR-TKIs (Kpuu ≤ 0.12) [23].

The ASCO-SNO-ASTRO guidelines state that local treatment of asymptomatic brain metastases with osimertinib can be postponed until CNS progression; therefore, osimertinib-fronted therapy is considered reasonable.

##### Dacomitinib

The second-generation EGFR-TKI dacomitinib is used as one of the standard therapies, and in the ARCHER1050 study, dacomitinib was superior to gefitinib as first-line therapy for EGFR mutation-positive NSCLC in PFS (14.7 m vs 9.2 m HR0.59; 95% CI 0.47–0.74 *p* < 0.001) and significantly prolonged OS (34.1 m vs 26.8 m HR0.760; 95% CI 0.582–0.993 *p* = 0.044) [24]. Jung et al. evaluated the intracranial efficacy of dacomitinib in NSCLC patients with brain metastases: 19 of 30 patients had intracranial CR and 10 had intracranial PR with an intracranial ORR of 96.7% (95% CI 80.9–99.8), indicating good intracranial tumor reduction. The intracranial ORR for dacomitinib was higher than the intracranial ORRs for gefitinib (65% and 68%), erlotinib (68%), and afatinib (73%) and similar to the intracranial ORR for osimertinib (91%). Twelve-month and 18-month dacomitinib intracranial PFS rates were 78.6% (95% CI 64.8–95.4) and 70.4% (95% CI 54.9–90.1), respectively, similar to the intracranial PFS rates for osimertinib (77% and 58%, respectively). It may be acceptable to administer dacomitinib initially for asymptomatic brain metastases and defer radiotherapy until symptoms appear [25].

##### Afatinib

The LUX-Lung3 study is a global phase III clinical trial that compared the efficacy and safety of 40 mg afatinib and cisplatin + pemetrexed in untreated patients with *EGFR* mutation-positive tumors. PFS was 11.1 and 6.9 months for afatinib and chemotherapy, respectively (HR 0.58; 95% CI 0.43–0.78; *p* < 0.01) [26]. The LUX-Lung6 study is a global phase III clinical trial that compared the efficacy and safety of 40 mg afatinib and cisplatin + gemcitabine in untreated patients with *EGFR* mutation-positive tumors. PFS was 11.0 and 5.6 months for afatinib and chemotherapy, respectively (HR 0.28; 95% CI 0.20–0.39; *p* < 0.0001) [27]. The LUX-Lung3 and LUX-Lung6 trials included 9.6% (35/345) and 12.6% (46/364) of the patients with brain metastases, respectively. In both trials, PFS was better with afatinib than with chemotherapy; however, the difference was not statistically significant (LUX-Lung3: afatinib 11.1 months, cisplatin + pemetrexed 5.4 months [HR HR 0.54; 95% CI 0.23–1.25; *p* = 0.1378]; LUX-Lung6: afatinib 8.2 months, cisplatin + gemcitabine 4.7 months [HR 0.47; 95% CI 0.18–1.21; *p* = 0.1060]). No significant differences were noted in OS of patients with brain metastases (Lux-Lung 3: afatinib 19.8 months, cisplatin + pemetrexed 33.2 months [HR 1.15; 95% CI 0.49–2.67; *p* = 0.7517]; Lux-Lung 6: afatinib 22.4 months, cisplatin + gemcitabine 24.7 months [HR 1.13; 95% CI 0.56–2.26; *p* = 0.7315]). A meta-analysis evaluating the efficacy of afatinib in patients with NSCLC and brain metastases has also been reported; 448 patients with brain metastases were divided into the control (chemotherapy and first-generation EGFR-TKIs) and afatinib groups. The HR for PFS was 0.58 (95% CI 0.39–0.85; *p* < 0.05), and for OS was 1.13 (95% CI 0.15–8.75; *p* > 0.05). Afatinib prolongs PFS, but not OS, compared with chemotherapy and first-generation EGFR-TKIs [28].

In contrast, a study on afatinib response rates in patients with brain metastases found that afatinib was significantly better than chemotherapy (Lux-Lung3: afatinib, 70.0%; cisplatin + pemetrexed, 20.0% [*p* = 0.0058]; Lux-Lung6: afatinib, 75.0%; cisplatin + gemcitabine, 27.8% [*p* = 0.0027]) [29]. The intracranial response rate was not determined in this study, and it remains unclear whether afatinib crosses the BBB and reaches sufficient concentrations in the CNS. Notably, in most patients, the brain is not the initial site of disease progression in most patients [28]. In a multicenter observational study examining cerebrospinal fluid penetration and the efficacy of afatinib in patients with NSCLC and oncogenic meningitis, CSF penetration was reported to be 2.4% on day 8 after the administration of 40 mg afatinib, indicating that afatinib migrated well into the CSF [30], suggesting a good penetration rate of afatinib into the central diameter.

##### Amivantamab-lazartinib chemotherapy

The MARIPOSA-2 study [31] compared PFS for amivantamab-lazertinib-chemotherapy, chemotherapy, and amivantamab-chemotherapy in patients with EGFR mutation-positive NSCLC whose disease progressed to osimertinib. PFS was significantly prolonged with amivantamab-chemotherapy and amivantamab-lazartinib chemotherapy compared to chemotherapy alone. [hazard ratio (HR) for disease progression or death 0.48 and 0.44, respectively; *p* < 0.001 for both; median of 6.3 and 8.3 versus 4.2 months, respectively]. Median intracranial PFS was 12.5 and 12.8 months for amivantamab-chemotherapy and amivantamab-lazartinib-chemotherapy, respectively, compared to chemotherapy (HR for intracranial disease progression or death 0.55 and 0.58, respectively).

Amivantamab and amivantamab-lazartinib chemotherapy improved PFS and intracranial PFS compared with chemotherapy alone. Although amivantamab is expected to be a large molecule that does not easily cross the blood–brain barrier, it is noteworthy that amivantamab chemotherapy demonstrated an advantage in intracranial PFS over chemotherapy, similar to amivantamab–lazartinib chemotherapy. Amivantamab improves intracranial PFS either through direct antitumor effects or indirectly through immune-based mechanisms.

##### Gefitinib plus chemotherapy

Hou et al. [32] conducted an open-label, prospective, multicenter, phase III randomized clinical trial investigating the efficacy and safety of gefitinib plus chemotherapy (pemetrexed and platinum) compared to gefitinib alone in untreated patients with EGFR-mutated NSCLC brain metastases. A total of 161 patients were enrolled and randomized to receive gefitinib (n = 81) or gefitinib plus chemotherapy (n = 80). Median intracranial PFS was 15.6 months (95% CI 14.3–16.9) in the gefitinib plus chemotherapy group versus 9.1 months (95% CI 8.0–10.2) in the gefitinib group (HR 0.36; 95% CI 0.25–0.53; *p* < 0.001). Similarly, median PFS was significantly longer for gefitinib plus chemotherapy than for gefitinib alone (16.3 months; 95% CI 14.4–18.2 vs 9.5 months; 95% CI 8.3–10.8; *p* < 0.001). Gefitinib plus chemotherapy had better intracranial objective response rates (85.0%; 95% CI 77.0–93.0 vs 63.0%; 95% CI 52.2–73.7; *p* = 0.002) and overall objective response rates (80.0%; 95% CI 71.0–89.0 vs 64.2%; 95% CI 53.5–74.9; *p* = 0.03). In this randomized clinical trial, gefitinib plus chemotherapy significantly improved intracranial PFS, PFS, and OS compared to gefitinib alone in patients with untreated EGFR-mutated NSCLC brain metastases, and may be an optional first-line therapy for these patients.

#### ALK tyrosine kinase inhibitors

The anaplastic lymphoma kinase (*ALK*) fusion gene is a gene translocation that accounts for 3–5% of NSCLC cases and was first reported in Japan in 2007 [33]. Brain metastases are present in 30% of patients at diagnosis and the cumulative incidence of brain metastases during the course of the disease is approximately 75%. Therefore, controlling brain metastases is important [34–36].

##### Alectinib

Second-generation ALK-TKI alectinib is recommended as a first-line therapy. In a phase I trial comparing alectinib with first-generation crizotinib (ALEX trial), alectinib significantly prolonged PFS and became the standard therapy (alectinib 34.1 months vs crizotinib 10.2 months; HR 0.37; 95% CI 0.26–0.52; p < 0.0001) [37, 38]. Alectinib is also highly effective in treating CNS diseases, with cumulative CNS progression rates of < 5% and approximately 10% at 18 months in patients with and without CNS disease, respectively, at baseline. CNS metastases are the most common sites of crizotinib progression. New lesions occurred in 70% and 20% of patients with and without brain metastases, respectively [39]. Crizotinib has low BBB permeability [40], whereas alectinib has high brain penetration and is not transported by the P-glycoprotein efflux transporter that maintains the BBB [41]. Thus, it was inferred that alectinib concentrations in the CNS were maintained. Alectinib appears to be an effective agent for the treatment of both systemic and intracranial diseases in patients with *ALK*-positive NSCLC. In a phase I/II clinical trial of alectinib in 47 patients with crizotinib-resistant *ALK*-positive NSCLC (AF-002JG), the CNS response rate was 77.8% (7/9 patients) in patients with measurable or non-measurable CNS disease at baseline, and the CNS disease control rate was 100% (all nine patients). Notably, four patients (44.4%) had a complete CNS response [42].

##### Brigatinib

Brigatinib, a second-generation ALK-TKI, significantly prolonged PFS in a phase I trial (ALTA-IL), as compared with crizotinib, as first-line therapy (brigatinib 24.0 months vs crizotinib 11.1 months; HR 0.48; 95% CI 0.35–0.66; p < 0.0001) and became a standardized therapy. Among the 81 patients with brain metastases at baseline (brigatinib, 40; crizotinib, 41), the probability of non-progression at 2 years was 43% (95% CI 2–59) for brigatinib and 10% (95% CI 2–25) for crizotinib (HR 0.25; 95% CI 0.14–0.46; *p* < 0.0001). In patients with measurable brain metastases, the intracranial response rates were 78% for brigatinib (14/18; 95% CI 52–94) and 26% for crizotinib (6/23; 95% CI 10–48). This indicates that brigatinib is effective in treating CNS metastases [43]. The duration of intracranial response was 27.9 and 9.2 months in the brigatinib (5.7 months to not estimable) and crizotinib (3.9 months to not estimable) groups, respectively, indicating that brigatinib significantly prolonged CNS response in the patients with measurable brain metastases at baseline. The 3-year intracranial PFS rate was 31% (95% CI 17–47%) in the brigatinib group and 9% (95% CI 2–25%) in the crizotinib group (HR 0.29; 95% CI 0.17–0.51; *p* < 0.0001), and better with brigatinib [44].

##### Lorlatinib

Lorlatinib, a third-generation ALK-TKI, also significantly prolonged PFS in a phase I trial (CROWN trial), compared with crizotinib, as first-line therapy and became standardized care (12-month progression-free rate, 78% lorlatinib vs 39% crizotinib; HR 0.28; 95% CI 0.19–0.41; *p* < 0.001). The 12-month CNS progression-free rate was also high (96%) [45]. Among the 78 patients with measurable or non-measurable CNS metastases at baseline, intracranial responses were achieved in 20% of patients treated with crizotinib (95% CI 9–36) and 66% of patients treated with lorlatinib (95% CI 49–80%), and were significantly higher with lorlatinib. Intracranial complete response rates were 61% and 15%, respectively. The percentages of patients with an intracranial response of 12 months or longer were 72% and 0%, respectively. Among the 30 patients with measurable CNS metastases at baseline, 82% (95% CI 57–96%) and 23% (95% CI 5–54%) had intracranial responses in the lorlatinib and crizotinib groups, respectively, and 71% and 8% had complete responses, respectively [46]. These results indicated that lorlatinib is effective in treating the brain metastases.

##### Ceritinib

ASCEND-4 is an open-label, randomized, phase III trial that compared the efficacy and safety of ceritinib- and platinum-based chemotherapy in patients with *ALK* translocation-positive NSCLC. PFS was 16.6 (95% CI 12.6–27.2) and 8.1 (95% CI 5.8–11.1) months in the ceritinib and chemotherapy groups, respectively, with a significant increase in the ceritinib group (HR 0.55; 95% CI 0.42–0.73; *p* < 0.00001). This study included 32.1% (121/376) of patients with brain metastases and 11.7% (44/121) of patients with active brain metastases. PFS for patients without brain metastases was significantly longer with ceritinib (HR 0.44; 95% CI 0.31–0.63): 25.2 months in the ceritinib group (95% CI 13.9–NE) and 8.3 months in the chemotherapy group (95% CI 5.8–11.1). PFS was also significantly longer for ceritinib in patients with brain metastases, 10.7 (95% CI 8.1–16.4) and 6.7 (95% CI 4.1–10.6) months in the ceritinib and chemotherapy groups at baseline, respectively (HR 0.70; 95% CI 0.44–1.12). The intracranial response rate in patients with measurable active brain metastases was 72.7% (16/22 patients: 2 complete and 14 partial responses) in the ceritinib group and 27.3% (6/22 patients: 2 complete and 4 partial responses) in the chemotherapy group, and was significantly higher in the ceritinib group [47].

The ASCO-SNO-ASTRO guidelines recommend alectinib, brigatinib, and ceritinib for *ALK*-positive NSCLC cases with asymptomatic brain metastases and state that local therapy can be postponed until intracranial progression when administered with these drugs.

#### ROS-1 tyrosine kinase receptor inhibitor

The c-ros oncogene1 (*ROS1*) fusion gene is a rare genetic translocation that accounts for approximately 1% of all NSCLC. Brain metastases are reported to occur in approximately 20% of patients at diagnosis, and 30–50% develop brain metastases during the course of the disease [47, 48].

##### Crizotinib

Several studies have reported the efficacy of crizotinib monotherapy in *ROS1* fusion-mutation-positive NSCLC. A 50-case study in the US showed a response rate of 72% (95% CI 58–84%) and a PFS of 19.2 months (95% CI 14.4–NE) [49]. In an additional study of 53 patients, OS was 51.4 months (95% CI 29.3–NE) [50]. In a 127-case study conducted in East Asia, the response rate was 71.7% (95% CI 63.0–79.3) and the PFS was 15.9 months (95% CI 12.9–24.0), both excellent results [51]. Patients with *ROS1*-positive NSCLC developed brain metastases (22.3%, 23/103) at the time of the initial diagnosis or recurrence. Patients without brain metastases developed brain metastases (30.0%, 24/80) during disease course. The time to the appearance of brain metastases was 12.0 months (95% CI 8.5–19.1). The most common site of metastasis for pemetrexed-based therapy was the extrathoracic region, whereas the most common site of metastasis for TKI therapy was the intracranial region (15.5%). Remarkably, intracranial progression was more common in patients treated with TKIs than in those treated with chemotherapy [52]. Costa et al. [38] reported that disease progression during crizotinib treatment was more likely to involve CNS metastases, including those in the brain and spinal cord. This may have been due to crizotinib resistance or poor transfer of crizotinib into the CSF [39]. Whole-brain radiation therapy, as a pretreatment for meningeal carcinomatosis, reportedly increases the CSF/plasma ratio [53]. A higher dose of 1000 mg/day was effective in treating metastatic brain tumors that developed during 500 mg/day crizotinib treatment [54]. Higher CSF levels may be more effective in treating CNS metastases. Therefore, crizotinib alone is ineffective at inhibiting CNS metastasis and should be combined with radiotherapy.

##### Entrectinib

The neurotrophic tropomyosin receptor kinase (NTRK) fusion gene is a driver mutation that is highly prevalent in salivary gland cancer, soft tissue sarcoma, and thyroid cancer in adults and is rarely present in colorectal cancer and small cell cancer [55]. NTRK genes include NTRK1, NTRK2, and NTRK3, which encode proteins of the tropomyosin receptor kinase (TRK) family (TRKA, TRKB, and TRKC) and function as transmembrane receptor tyrosine kinases. Entrectinib is a drug that inhibits TRKA, TRKB, TRKC, ROS1, and ALK, and following its approval for NTRK fusion gene-positive solid tumors, it has also been approved for ROS-1 fusion gene-mutated NSCLC. Targeted agents with activity in the central nervous system are needed because 40% of patients with ROS1 fusion-positive metastatic NSCLC have central nervous system metastases at the time of diagnosis. Entrectinib is designed to cross the blood–brain barrier [56].

Integrated analyses of phase I and II trials (ALKA-372-001, STARTRK-1, and STARTRK-2) were conducted for entrectinib monotherapy in *ROS1* fusion mutation-positive NSCLC. In the updated 161-patient study, the response rate was 67.1% (95% CI 59.3–74.3) and the PFS was 15.7 months (95% CI 11.0–21.1). In 24 patients with CNS metastases at baseline, the intracranial response efficiency was 79.2% (19/24 patients; 95% CI 57.9–92.9) and intracranial PFS was 12.0 months (95% CI 6.2–19.3). These results are favorable [57].

##### Repotrectinib

Similar to entrectinib, repotrectinib has been developed to penetrate the BBB. Lepotrectinib may also be effective in ROS1 mutation-positive patients with brain metastases. Repotrectinib has shown promising efficacy in patients with ROS1-positive NSCLC in a subgroup analysis of the TRIDENT-1 trial. The study included seven patients with measurable BM at baseline; three TKI-naïve patients had an intracranial ORR of 100%, and four pre-TKI patients had an intracranial ORR of 50%. The mDoR for both groups was 5.5 months [58].

#### BRAF inhibitor

The v-raf murine sarcoma viral oncogene homolog B1 (*BRAF*) mutation is a rare genetic mutation that accounts for 1–3% of NSCLC cases [16]. Combination therapy with the BRAF inhibitor dabrafenib and the MEK inhibitor trametinib is effective for treating *BRAF* V600E mutation-positive NSCLC. In previously treated patients, the response rate was 68.4% (95% CI 54.8–80.1), PFS was 10.2 months (95% CI 6.9–16.7), and OS was 18.2 months (95% CI 14.3–28.6). In untreated patients, the response rate was 63.9% (95% CI 46.2–79.2), PFS was 10.8 months (95% CI 7.0–14.5), and OS was 17.3 months (95% CI 12.3–40.2) [59]. No studies have described the effect of dabrafenib/trametinib on *BRAF* V600E mutation-positive NSCLC with brain metastases, and its effects on brain metastases remain unknown.

#### MET inhibitor

Exon 14 mutations in the mesenchymal-to-epithelial transition (MET) gene account for approximately 3–4% of NSCLC cases [60, 61].

##### Tepotinib

The VISION trial is a phase II single-arm study investigating the efficacy and safety of the MET inhibitor tepotinib in NSCLC patients with MET mutations (exon 14 skipping) or amplification. The response rate was 46.0% (95% CI 36–57), DoR was 11.1 months (95% CI 7.2–NE), PFS was 8.5 months (95% CI 6.7–11.0), and OS was 17.1 months (95% CI 12.0–26.8), with favorable results [62]. In 11 patients with brain metastases, the response rate was 55% (95% CI 23–83), the DoR was 9.5 months (95% CI 6.6–estimable), and the PFS was 10.9 months (95% CI 8.0–estimable), with good results [63]. In a subgroup analysis of the VISION study, 13 of 15 patients with brain metastases showed disease control, and five of seven patients with measurable disease showed a partial response. In addition, in a report of a lung adenocarcinoma patient with MET ex 14 del using tepotinib as fifth-line therapy, symptoms disappeared on day 10 and brain metastasis disappeared on head MRI on day 23 [64]. In addition, a patient with lung adenocarcinoma and meningeal dissemination of the MET exon 14 skipping mutation was treated with tepotinib as a second-line therapy, which showed a reduction in meningeal dissemination and an improvement in PS. Tepotinib reached a plasma concentration of 1648 ng/mL and CSF concentration of 30.6 ng/mL, with a good penetration rate (CSF/plasma) of 1.83% [65]. Based on these findings, tepotinib may be effective against brain metastases from lung adenocarcinoma with MET exon 14 skipping mutation. In a preclinical study, the total tepotinib concentration was reported to be 2.87 × higher in the brain than in the plasma after intravenous administration of tepotinib to three Wistar rats. The calculated unbound brain–plasma ratio was 0.25, indicating adequate brain penetration [66]. We believe that effective brain penetration contributes to these effects in the CNS.

##### Capmatinib

Dagogo-Jack et al. [67] conducted a phase II study to evaluate the efficacy of capmatinib in patients previously treated with MET inhibitors. Twenty patients (15 and five patients with MET skipping alterations and amplification, respectively) were previously treated with MET inhibitors; two patients achieved an objective response, 14 patients had stable disease, and the disease control rate was 80%. PFS was 5.5 months (95% CI 1.3–11.0) and OS was 11.3 months (95% CI 5.5–unachieved). The CNS disease control rate was 100% in four patients with measurable brain metastases. Wolf et al. [68] conducted a multi-cohort phase II trial to evaluate capmatinib in patients with advanced NSCLC and MET dysregulation. Patients were assigned to cohorts based on their prior line of therapy and MET status (MET exon 14 skipping mutations or amplifications). In NSCLC patients with MET exon 14-skipping mutations, an overall response was observed in 41% (95% CI 29–53) of 69 patients who were previously treated with one or two lines, and in 68% (95% CI 48–84) of 28 patients who were previously untreated. DoR was 9.7 months (95% CI 5.6–13.0) and 12.6 months (95% CI 5.6–NE), respectively. In the same study, 12 of 13 patients (10 previously treated and 3 previously untreated) with brain metastases and MET exon 14 skipping mutations showed intracranial responses, including four complete responses. Based on these findings, capomatinib may be effective against CNS metastases in patients with NSCLC harboring MET exon 14 skipping mutations.

#### RET inhibitor

The RET proto-oncogene encodes the transmembrane receptor tyrosine kinase (RET), and chromosomal rearrangements of the RET gene result in abnormal RET expression in cells that are not normally transcribed, leading to the production of oncogenic intracellular RET fusion proteins in a ligand-independent manner. RET fusions are present in 1–2% of NSCLC cases and represent important new therapeutic targets [69].

##### Selpercatinib

The LIBRETTO-001 trial was a phase I/II, single-arm, open-label study of selpercatinib in patients with RET-fusion-positive NSCLC, including 69 treatment-naïve patients and 247 patients previously treated with platinum-based chemotherapy. Among treatment-naïve patients, the overall response rate was 84% (95% CI 73–92), with 6% achieving a complete response; DoR was 20.2 months (95% CI 13.0–NE), and PFS was 22.0 months. In patients previously treated with platinum-based chemotherapy, the ORR was 61% (95% CI 55–67) and 7% achieved a complete response, DoR was 28.6 months (95% CI 20.4 to not evaluable), and PFS was 24.9 months. In 26 patients with measurable CNS metastases at baseline, the intracranial response rate was 85% (95% CI 65–96), and 27% had a complete response. Selpercatinib shows a sustained and robust response in intracranial disease [70]. Subbiah et al. [71] evaluated the intracranial response rate in 80 patients with brain metastases at baseline in the LIBRETTO-001 trial by using MRI/CT every eight weeks for one year. Twenty-two patients had measurable brain metastases. The intracranial response rate was 82% (95% CI 60–95), and 23% had a complete response. The intracranial PFS was 13.7 months (95% CI 10.9–NE). Selpercatinib exerts strong and durable intracranial effects in patients with RET fusion-positive NSCLC.

##### Pralsetinib

ARROW [72] is a multi-cohort, open-label, phase I/II study evaluating the safety, tolerability, and antitumor activity of pralsetinib, a highly active oral selective RET inhibitor, in patients with RET fusion gene positive NSCLC. Preclinical studies on pralsetinib have demonstrated its ability to penetrate the blood–brain barrier and its activity against intracranial tumors. In this study, a reduction in intracranial metastases was observed in all nine patients with measurable intracranial metastases at baseline and at least one post-baseline intracranial response assessment. Five of nine patients (56%; 95% CI 21–86) had an intracranial response, including three patients with complete responses, and Kaplan–Meier estimate of the probability of continued intracranial response at 6 months was 80% (95% CI 45–100), and 53% (95% CI 5–100) at 12 months. In this study, pralcetinib demonstrated intracranial activity, including induction of a complete intracranial response, and is likely to be useful in patients with brain metastases.

#### KRAS genetic variant

*KRAS* mutations are found in approximately 30% of lung adenocarcinomas in Europe and the US, and *KRAS* G12C mutations account for 41% of all *KRAS* mutations [73]. Selective KRAS inhibitors, such as sotorasib, are effective against *KRAS* G12C mutation-positive lung cancer [74]. Sotorasib was the first drug approved for *KR*AS G12C-mutant NSCLC, and compared to docetaxel, the response rates were 28.1% (95% CI 21.5–35.4) vs 13.2% (95% CI 8.6–19.2; *p* < 0.001), disease control rates were 82.5% (95% CI 75.9–87.8) vs 60.3% (95% CI 52.7–67.7), and the PFS was 5.6 months (95% CI 4.3–7.8) vs 4.5 months (95% CI 3.0–5.7), respectively. Brain metastasis recurrence occurred later with sotorasib than with docetaxel in patients with brain metastases (15.8 months [95% CI 9.7–NE] vs10.5 months [95% CI 5.8–NE]). Therefore, sotorasib may be effective in the treatment of systemic diseases and central metastases [75]. Sotorasib was used in four treatment-naïve patients with active brain metastases and measurable disease, with an intracranial response rate of 75% (3/4), PFS of 4.1 months (95% CI 3.9–NE), and CNS-PFS of 4.7 months (95% CI 3.9–NE) [76]. Further studies on the efficacy of sotorasib against patients with brain metastases are required.

Vassella et al. [77] genetically evaluated 54 pathological specimen pairs of brain metastases and primary lung cancer. Nineteen percent (10/54) of the cases had the same set of mutations in the primary and metastatic sites, with no evidence of private mutations. TP53, KRAS, and MYC mutations were the most frequent in this group. Twenty-two percent of the specimens (12/54) showed private mutations at the primary site, with RICTOR being the most frequent private mutation. 26% of the specimens (14/54) had private mutation at the brain metastasis. 26% of the specimens (14 of 54) had private mutations in both the primary site and brain metastasis site. Private mutations at metastatic sites frequently had additional KRAS mutations (21%) and KRAS copy number gains (21%). In contrast, only one private KRAS alteration was found at the primary site (1of 54). In 7% of the specimens (4/54), the tumor pairs were pure lung adenocarcinomas with no mutations shared between the primary site and the brain metastatic site. This group may be composed of collision tumors. In addition, 50% (14 of 28) of the samples with private mutations at the brain metastasis site had mutations in genes involved in the EGFR signaling pathway (activating mutations (n = 3), copy number alterations (CNA) of the KRAS gene (n = 3), activating mutations or CNA in PIK3CA (n = 2), CNAs in MET (n = 2), RICTOR (n = 2), AKT2 (n = 1), EGFR Exon 2–7 skipping variant EGFRvIII (n = 1), and NF1 truncating mutations (n = 2). In summary, more than half of tumors with private mutations at brain metastatic sites have various types of genetic mutations involving the EGFR signaling pathway, suggesting that this pathway may be particularly important at metastatic sites.

### Antibody–drug conjugate (ADC)

HER2 is expressed on the surface of many cancer cells, including lung, breast, stomach, and colon cancer cells, and is involved in cell growth. HER2 mutations are found in approximately 2–4% of NSCLC [78]. A higher rate of brain metastasis has been noted in HER2 mutant lung cancer than in lung cancers with other driver mutations [79]. ADCs have been developed as molecular-targeted drugs against HER2. ADCs combine an antibody with a drug (a low-molecular-weight compound) via an appropriate linker to deliver the drug directly to cancer cells via an antibody that binds to a target factor expressed on cancer cells, thereby reducing systemic exposure to the drug and enhancing its ability to attack cancer cells. This reduces the systemic exposure to drugs and increases their ability to attack cancer cells. Trastuzumab deruxetecan, an anti-HER2 antibody drug conjugate, and patritumab deruxetecan, an anti-HER3 antibody drug conjugate, have been shown to be effective against NSCLC and are taken into cells by binding to HER2 or HER3 expressed on the cell membrane of tumor cells, and once the linker is hydrolyzed, When the linker is hydrolyzed, camptothecin derivatives and U3-1402 are released, causing DNA damage and inducing apoptosis, thereby exerting an antitumor effect.

#### Trastuzumab deruxecan

The DESTINY-Lung01 Study [80] was a multicenter, international phase II trial of trastuzumab deruxecan (6.4 mg per kg body weight) in patients with metastatic HER2-overexpressing or HER2 mutated NSCLC refractory to standard therapy; ORR was 55% (95% CI 44–65) (50 of 91), with CR in one patient. The disease control rate was 92% (95% CI 85–97) with tumor shrinkage in most patients; PFS 8.2 months (95% CI 6–11.9) and OS 17.8 months (95% CI 13.8–14.7) were excellent. Patients with CNS metastases at baseline also showed good antitumor efficacy with a response rate of 54% (18 of 33), PFS of 7.1 months (95% CI 5.5–9.8) and OS of 13.8 months (95% CI 9.8–20.9). DESTINY-Lung02 [81] was a blinded, multicenter, phase II trial of trastuzumab deruxetequan (5.4 mg and 6.4 mg per kg body weight) in patients with treatment-naive NSCLC with metastatic HER2 mutations. 35 (34.3%) and 22 (44.0%) patients in the 5.4 and 6.4 mg arms had stable CNS metastases at baseline. ORR was 49% (95% CI 39–59.1) and 56% (41.3–70.0), respectively, with a median duration of response of 16.8 months (95% CI 6.4–NE) and NE (8.3–NE) PFS was 9.9 months (95% CI 7.4–NE) and 15.4 months (8.3–NE), and OS was good at 19.5 months (95% CI 13.6–NE) and NE (12.1–NE). In patients with CNS metastases at baseline, the ORR in the 5.4 mg group was 60.0% (95% CI 42.1–76.1) in patients with brain metastases and 43.3% (31.2–56.0) in patients without brain metastases. The ORR in the 6.4 mg group was 45.5% (95% CI 24.4–67.8) in patients with brain metastases and 63.3% (44.1–81.4) in patients without brain metastases, and responses were observed in the entire treatment group.

#### Patritumab deruxtecan

The HERTHENA-Lung01 trial [82] was a phase II study evaluating patritumab deruxetecan as a third-line therapy in 225 patients with EGFR mutation-positive metastatic or locally advanced NSCLC whose disease had progressed after EGFR-TKI plus platinum-based combination therapy. ORR was 29.8% (95% CI 23.9–36.2) (one complete response and 66 partial responses), DOR 6.4 months (95% CI 4.9–7.8), DCR 73.8%, PFS 5.5 months (95% CI 5.1–5.9), OS 11.9 months (95% CI 11.2–13.1) The results were as follows. In an analysis of 30 patients with brain metastases without prior baseline radiotherapy, the CNS ORR was 33.3% (95% CI 17.3–52.8) (nine complete responses and one partial response) and the median duration of response for intracranial response was 8.4 months. The efficacy ofintracranial therapy was excellent.

## Immune checkpoint inhibitor

Immune checkpoint inhibitors (ICIs) are key drugs for driver mutation-negative lung cancer, and PD-Ll expression in tumors is a predictor of anti-PD-1/PD-L1 antibody efficacy. The concordance of PD-L1 expression between the primary tumor and brain metastases, as well as the penetration of the BBB by anti-PD-1/PD-L1 antibodies and activated T lymphocytes, is important in predicting the efficacy of ICI.

### PD-L1 expression in primary tumor and brain metastases

Mansfield et al. [83] reported PD-L1 expression in primary lung cancer and brain metastases in 146 paired specimens collected from 73 patients. Fifty-six patients (39%) were PD-L1 positive. These included 32 primary lung cancer (44%) and 24 brain metastases (33%) cases. PD-L1 expression in the primary lesions and brain metastases was concordant in 63 cases (86% overall; 95% CI 76–93) and discordant in 10 cases (14% overall; 95% CI 7–24). Wei et al. [84] reviewed 10 studies on the efficacy of ICI treatment for intracerebral and extracerebral lesions. The intracerebral response rate was approximately 30%, which was similar in each study, and approximated the response rate of the primary lesion. The intracerebral disease control rate was approximately 50%, similar to the systemic disease control rate, and the treatment efficacy of ICIs for brain metastases and extracerebral lesions was similar. The antitumor efficacy of ICIs for CNS metastases and primary lung lesions was inconsistent in four of the six studies (87 cases in total) that investigated the efficacy of ICIs against CNS metastases and primary lung lesions. In a pooled analysis, the concordance rate was 80.5% (70/87 and the discordance rate was 19.5% (17/87); of the 17 discordant cases, nine (10.3%) responded only to intracranial lesions and eight (9.2%) to extracranial lesions. In many patients, the antitumor effect of ICIs is concordant with primary lung and brain metastases; however, in a few patients, the sensitivity to ICIs differs between primary lung and brain metastases.

### Central migration of ICI

Although the permeability of substances is tightly controlled at the BBB and protein concentrations in the CSF are maintained at low levels, brain metastasis can disrupt the BBB and alter its permeability.

Pluim et al. [85] measured the concentration of PD-1 inhibitors in CSF using an enzyme-linked immunosorbent assay. The serum/CSF ratio ranged from 52 to 299, and the permeability was poor. The effect of ICIs on brain metastases is believed to depend on activated immune cells and not on CSF permeability, which alters the tumor microenvironment by activating the host immune system, enhancing antigen presentation, and modulating cytokines in the host body to improve the recognition and aggressiveness of tumor cells by the immune system. In contrast, ICIs enhance the efficacy of radiotherapy by modulating the tumor microenvironment, normalizing the tumor vasculature, and improving tumor hypoxia. The combination of radiotherapy and ICI has been shown to produce synergistic antitumor activity. In addition, intracranial radiation therapy increases BBB permeability, allowing ICIs to enter brain tissue [86]. Next, we discuss the clinical effects of ICI treatment on brain metastases.

### Clinical results of ICI

Although patients with brain metastases at baseline are often excluded from clinical trials, several studies have included them KEYNOTE-189 is a phase III trial evaluating the efficacy of pembrolizumab plus chemotherapy in untreated advanced recurrent non-squamous NSCLC. A total of 108 patients with brain metastases were enrolled in this study. In a subgroup analysis of patients with brain metastases, OS was 19.2 months (95% CI 15.0–25.9) in the pembrolizumab + chemotherapy group and 7.5 months (95% CI 4.6–10.0) in the chemotherapy group (HR 0.41; 95% CI 0.24–0.67). In the group without brain metastases, OS was 22.4 months (95% CI 19.7–24.5) vs 12.1 months (95% CI 9.1–15.0), respectively (HR 0.59; 95% CI 0.46–0.75). Pembrolizumab plus chemotherapy, with or without brain metastases, has been shown to prolong survival, similar to PFS [87].

KYENOTE 024 is a study comparing pembrolizumab monotherapy with chemotherapy as first-line therapy in patients with a PD-L1 TPS ≥ 50%. A total of 9.1% (28/305) of patients with brain metastases were enrolled. In a subgroup analysis of PFS in patients with brain metastases, 17 of 28 patients showed disease progression (HR 0.55; 95% CI 0.20–1.56) with pembrolizumab vs chemotherapy [88]. The OAK trial compared the efficacy of atezolizumab with that of docetaxel as second-line therapy, regardless of PD-L1 expression. The study enrolled 10% (85/850) of patients with brain metastases, and OS for patients with brain metastases was 20.1 and 11.9 months for atezolizumab and docetaxel, respectively (HR 0.54; 95% CI 0.31–0.94). OS in patients without brain metastases was 13.0 and 9.4 months, respectively (HR 0.75; 95% CI 0.63–0.89), indicating that atezolizumab extended OS better then docetaxel in patients with and without brain metastases [89]. The IMpower150 trial randomized 1202 chemotherapy-naïve patients with nonsquamous NSCLC receiving atezolizumab + bevacizumab + carboplatin + paclitaxel (ABCP), atezolizumab + carboplatin + paclitaxel (ACP), or bevacizumab + carboplatin + paclitaxel (BCP) therapies and evaluated efficacy. A subgroup analysis of patients with brain metastases investigated the incidence of new brain metastases and time to onset. In total, 8.3% (100/1202) of patients developed new brain metastases at the data cutoff. New brain metastases occurred in 7.0% (28/400), 11.9% (48/402), and 6.0% (24/400) of patients in the ABCP, ACP, and BCP groups, respectively. The HR point estimates for time to develop new brain metastases were 0.68 for ABCP (95% CI 0.39–1.19) versus BCP. Therefore, ABCP cannot reduce the incidence of new brain metastases compared to BCP but may increase the time to the development of brain metastases [90].

Pooled analyses also examined the efficacy of ICIs in patients with brain metastases. The KEYNOTE series of clinical trials (KEYNOTE 021, 189, and 407) showed that pembrolizumab plus chemotherapy prolonged survival better than chemotherapy alone, regardless of the presence of brain metastases at baseline. Overall, 13% (171/1298) of patients had brain metastases at baseline. OS in patients with brain metastases was 18.8 months with pembrolizumab + chemotherapy vs 7.6 months with chemotherapy (HR 0.48; 95% CI 0.32–0.70) and PFS was 6.9 months vs 4.1 months (HR 0.44; 95% CI 0.31–0.62). These results were not significantly different from those of patients without brain metastases, suggesting the benefit of pembrolizumab + chemotherapy in patients with brain metastases [91]. Several meta-analyses have reported the efficacy of ICIs in patients with brain metastases. Chu et al. [92] conducted a network meta-analysis in a prospective study to compare the efficacy of various ICI therapies in patients with NSCLC and brain metastases. ICI (HR 0.76; 95% CI 0.62–0.92), ICI + chemotherapy (HR 0.44; 95% CI 0.33–0.58), ICI + ICI (HR 0.65; 95% CI 0.45–0.94), and ICI + ICI + chemotherapy (HR 0.4; 95% CI 0.3–0.54) prolonged OS. ICI + ICI + chemotherapy and ICI + chemotherapy showed the best OS rates. ICI (HR 0.83; 95% CI 0.66–1), ICI + chemotherapy (HR 0.43; 95% CI 0.31–0.59), and ICI + ICI + chemotherapy (HR 0.4; 95% CI 0.25–0.64) extended PFS better than chemotherapy. ICI + ICI + chemotherapy and ICI + chemotherapy demonstrated the best PFS. Given the prolonged OS and PFS, ICI + ICI + chemotherapy and ICI + chemotherapy may be clinically effective, and these regimens should be considered for patients who are negative for driver mutations. CheckMate 227 Part 1 [93] showed that nivolumab and ipilimumab prolong OS compared with chemotherapy in patients with metastatic NSCLC, regardless of tumor programmed death ligand 1 (PD-L1) expression. Reck et al. reported the efficacy of intracranial therapy in patients with brain metastases at baseline during a five-year follow-up period. Of the 1739 patients, 202 had baseline brain metastases (nivolumab plus ipilimumab: 68 patients; chemotherapy: 66 patients). Nivolumab and ipilimumab prolonged OS compared to chemotherapy in patients with brain metastases HR 0.63; 95% CI 0.43–0.92) and in patients without brain metastases (HR 0.76; 95% CI 0.66–0.87) The five-year survival rate and intracranial progression-free survival rate were higher for nivolumab plus ipilimumab (12% and 16%, respectively) than for chemotherapy (0% and 6%). Fewer patients treated with nivolumab plus ipilimumab developed new brain lesions among patients with brain metastases at baseline (4%) than among those treated with chemotherapy (20%). The efficacy of ICI in patients with brain metastases was also confirmed in CheckMate 817 [94], which examined the efficacy of first-line nivolumab and ipilimumab in metastatic NSCLC, including patients with PS2 and asymptomatic brain metastases. Overall, 391 patients were validated, and 49 patients with inactive brain metastasis were included. The median OS (95% CI) was 16.8 months (14.6–22.4), three-year OS rate (95% CI) was 33.7% (29.0–38.5%), median PFS (95% CI) was 5.8 months (4.5–7.6), three-year PFS rate (95% CI) was 20.1% (15.9–24.7%) The ORR (95% CI) was 20.1% (15.9–24.7%). The ORR (95% CI) was 37.3% (32.5–42.3%), and the median DOR (95% CI) was 27.6 months (20.4–34.3). Forty-one percent (32–50%) of the responders remained responsive after three years. For untreated patients with brain metastases, median OS (95% CI) was 12.8 months (7.7–25.9), three-year OS rate (95% CI) was 21.0% (10.9–33.4%), median PFS (95% CI) was 2.8 months (1.7 to 8.0), three-year PFS rate (95% CI) was 14.2% (5.4% to 27.1%). The ORR (95% CI) was 0.9 (1.7–1.0). The ORR (95% CI) was 32.7% (19.9–47.5%), and the median DOR (95% CI) was 12.6 months (6.7–NE). 39% (15–64%) of responders remained responsive after three years.

The ASCO-NSO-ASTRO guidelines recommend that patients with asymptomatic brain metastases and immunotherapy-naïve, PD-L1-positive NSCLC should consider platinum + pemetrexed + pembrolizumab and recommend a multifaceted discussion of the risks and benefits of local therapy. Further information regarding the efficacy of ICI in patients with brain metastases is required.

### Combination of ICI and radiotherapy

Although ICIs have dramatic and long-lasting effects in some patients with lung cancer, many respond poorly to treatment. Although PD-L1 expression generally correlates with ICI response, some patients with low PD-L1 expression may also benefit from ICIs treatment, suggesting the presence of factors other than PD-L1 expression. Strategies to combine ICIs with other therapies have been investigated to improve response rates to ICIs, and combinations of cytotoxic T-lymphocyte-associated protein 4 (CTLA-4) antagonists and radiation therapy have been shown to improve efficacy in clinical practice.In the five-year follow-up data of the Checkmate 227 trial, five-year survival rates were 24% for nivolumab + ipilimumab and 14% for nivolumab in the PD-1 ≥ 1% population; in the PD-1 < 1% population, the 5-year survival rates were 19% and 7%, respectively. The addition of the CTLA-4 antagonist ipilimumab to the anti-PD-1 antibody nivolumab increased survival rates [95] and increased awareness of optimal therapy combined with PD-1/PD-L1 antibodies. The PACIFIC trial was a phase III study that evaluated PFS and OS with PD-L1 antibody durvalumab in patients with unresectable Stage III NSCLC that did not progress after concurrent chemoradiation therapy (CCRT). Durvalumab prolonged PFS better than the placebo (durvalumab 16.9 months vs placebo 5.6 months; HR 0.52; 95% CI 0.42–0.65; p < 0.001) [96]. The combination of radiotherapy and ICI can control local diseases and improve survival rates.

El Rassy et al. [97] performed a comprehensive review of the use of ICI in patients with NSCLC and brain metastases and described the efficacy of combining ICI and radiation therapy. Preclinical studies have shown that low-dose fractionated radiotherapy leads to upregulation of tumor cell PD-L1 in neogenic mouse models of cancer. The combination of anti-PD-1/PD-L1 antibody and fractionated radiation therapy also elicited CD8 + T cell responses that improved local tumor control, long-term survival, and maintenance of protection against tumor rechallenge. IFNγ produced by CD8 + T cells is involved in PD-L1 upregulation in tumor cells after radiation therapy. The concurrent administration of fractionated radiotherapy is necessary to improve patient survival. In particular, the scheduling of PD-L1 antibodies has been shown to be important for therapeutic efficacy [98]. Radiation has been reported to enhance the efficacy of ICIs in clinical practice. In a secondary analysis of the KEYNOTE-001 trial, Shaverdian et al. [99] evaluated the PFS, OS, and safety in patients with and without radiation therapy for NSCLC before pembrolizumab treatment; 42 of 97 patients (43%) received radiation therapy. The median PFS was 4.4 months (95% CI 2.1–8.6) for patients previously treated with radiation and 2.1 months (95% CI 1.6–2.3) for radiation-naïve patients. The median OS was 10.7 months (95% CI 6.5–18.9) vs 5.3 months (95% CI 2.7–7.7), respectively, suggesting that the combination of ICI and radiation may be effective.

Altan et al. [100] conducted a phase I/II physician-initiated trial of nivolumab and ipilimumab in combination with SRS for active brain metastasis of NSCLC to evaluate safety and four-month intracranial progression-free survival (PFS). Thirteen patients were enrolled, 10 of whom were evaluated for dose-limiting toxicity (DLT). Three patients, in addition to one patient with DLT, experienced grade 3 or higher adverse events, including elevated liver function test results, fatigue, nausea, adrenal insufficiency, and myocarditis. One patient died seven months after the initiation of the protocol therapy (outside the DLT evaluation period) due to influenza infection, pneumonia, and hemophagocytic lymphohistiocytosis. The estimated four-month intracranial PFS rate was 70.7%. Brain SRS with nivolumab plus ipilimumab was safe for patients with active NSCLC brain metastases, and preliminary analysis of the treatment response showed that it promoted intracranial treatment response.

Clinical Trials.Gov, a database of clinical trials and research, has shown that several clinical trials of combined ICI and radiation therapy are currently underway (Table 1), suggesting that radiation therapy may further improve the therapeutic effect of ICI treatment.

**Table 1:** Ongoing clinical trials combining ICI and radiation therapy

	Target	Exam status	Study arm	Outcome measure (primary)	Symptoms of brain metastases	N	Study type	
NCT05522660	NSCLC, Melanoma	RECRUITING	ICI or Targeted therapy	CNS-specific PFS	Asymptomatic, Oligo-symptomatic	190	Interventional	RCT Phase III
			ICI or Targeted therapy + SRS					
NCT04835025	NSCLC	SUSPENDE	Radiotherapy	progression-free surival	–	200	Observational	Retrospective Controlled Study
			Radiotherapy + ICI					
NCT05638425	NSCLC	RECRUITING	Radiotherapy + PD-1 inhibitors	Intracranial clinical benefit rate, PFS	Symptomatic	20	Observational	Prospective Single arm
NCT05703269	NSCLC, Renal cell carcinoma, Breast Carcinoma, Melanoma	RECRUITING	ICI + single fraction stereotactic radiosurgery (SSRS)	Occurrence of a Grade 2 or higher Adverse Radiation Effect	–	244	Interventional	RCT Phase III
			ICI + fractionated stereotactic radiosurgery (FSRS)					
NCT02858869	NSCLC, Melanoma	ACTIVE, NOT RECRUITING	Pembrolizumab + SRS 6 Gy	Proportion of dose limiting toxicities	–	27	Interventional	Pilot Study Phase I
			Pembrolizumab + SRS 9 Gy					
			Pembrolizumab + SRS 18-21 Gy					
NCT02696993	NSCLC	RECRUITING	Nivolumab + SRS	Recommended phase 2 dose (RP2D) of nivolumab (Phase I) RP2D of nivolumab in combination with ipilimumab (Phase I) PFS (Phase II)	–	88	Interventional	Phase I Phase II Single arm
			Nivolumab + WBRT					
			Nivolumab + ipilimumab + SRS					
			Nivolumab + ipilimumab + WBRT					

## Conclusion

This review outlines the efficacy of molecular-targeted agents and immune checkpoint inhibitors in the treatment of brain metastases from lung cancer. Although local therapy, such as surgery and radiotherapy, is the basic treatment for brain metastases, it may not be optimal in some cases, depending on the general condition of the patient, owing to its invasiveness and risk of late adverse events. Data on drug therapies, such as molecular-targeted drugs and ICIs, which are effective for brain metastases are accumulating, and new research is underway on the combination of ICIs and radiotherapy. Determining the optimal treatment for each patient is necessary to improve the prognosis, QOL, and patient satisfaction. Multidisciplinary treatment by oncologists, respiratory physicians, neurosurgeons, radiologists, and radiation oncologists has become increasingly important for applying the latest information in clinical practice.

## Data Availability

None.
